# Why Current EU Proposals for Corona-Related Financial Aid Cannot Replace Coronabonds

**DOI:** 10.1007/s10272-020-0892-2

**Published:** 2020-06-07

**Authors:** Sebastian Dullien, Thomas Theobald, Silke Tober, Andrew Watt

**Affiliations:** Macroeconomic Policy Institute (IMK), Hans-Böckler-Straße 39, 40476 Düsseldorf, Germany

## Abstract

With public debt-to-GDP levels now set to surpass post-war records and Italy’s ratio approaching levels reached in Greece on the eve of the country’s debt restructuring in early 2012, fears of a return of the sovereign debt crisis have emerged.

Across Europe, the coronavirus crisis has pushed economies into recessions of a depth not experienced in generations. The collapse in economic activity in turn is delivering a triple blow to public debt ratios. First, automatic stabilisers have led to a surge in deficits as revenues have collapsed and payments for income support skyrocketed. Second, discretionary measures to stabilise the economy against the deep shock are adding hundreds of billions of unfunded expenditure and targeted tax relief to public deficits. Third, collapsing GDP has led to a further increase of debt ratios by mere arithmetic: in the case of Italy's public debt of 135% of GDP in 2019, a drop in nominal GDP by 10% in itself increases the debt-to-GDP level by 15 percentage points.

**Sebastian Dullien**, Macroeconomic Policy Institute (IMK), Düsseldorf; and HTW Berlin – University of Applied Sciences, Germany.

**Thomas Theobald**, University of Bamberg; and Macroeconomic Policy Institute (IMK), Düsseldorf, Germany.

With public debt-to-GDP levels now set to surpass post-war records and Italy's ratio approaching levels reached in Greece on the eve of the country's debt restructuring in early 2012, fears of a return of the sovereign debt crisis have emerged. The combination of weak economic activity, large fiscal deficits and high debt levels eerily resembles the situation in 2010, when investors panicked amid a sudden increase in reported Greek debt levels and triggered the euro crisis. At that time, first Greece and then several other euro countries successively experienced a substantial increase in their sovereign risk premiums. This gave rise to a vicious cycle of rising public borrowing costs, plunging economic activity, capital flight and further soaring risk premiums (Theodoropoulou and Watt, 2012). The role of sovereign yields as benchmarks for private lending rates amplified the economic contraction (Iorgova et al., 2012; Gorton, 2017; Theobald and Tober, 2020).

Until the summer of 2012, the efforts by euro area governments, involving several rescue funds amounting to a total of almost €1 billion in lending capacities coupled with harsh austerity measures, failed to calm financial markets. The rescue measures lacked conviction as they represented a minimalist consensus arising from a drawn-out and costly struggle between national interests rather than a convincing European answer. Only when European Central Bank (ECB) president Mario Draghi issued the famous promise to do "whatever it takes" to save the euro, did the panic subside and the spreads narrow.

## A renewed increase in government spreads

In the current crisis, the European Commission has suspended the fiscal rules and eased restrictions on state aid to companies. The ECB has once again, after initial hesitation, acted resolutely with the announcement of the purchase of €750 billion in government bonds – with an option for more – under the Pandemic Emergency Purchase Programme (PEPP). Yet an open question remains: How long can the ECB keep spreads from rising? The May 2020 ruling of the German constitutional court questioning the legality of the ECB's Public Sector Purchase Programme (PSPP) in particular has raised new concerns about possible limits to the central bank's policies (Brade and Gentzsch, 2020).

Moreover, the marked renewed increase in government spreads despite the ECB's interventions is putting further pressure on the European single market, already weakened by the uncoordinated closure of borders between EU member states and initial export restrictions of medical supplies on the part of some member states. With rising spreads, interest rates increase not only for newly issued government debt, but for the entire national economy through valuation and benchmark effects (Theobald and Tober, 2020). As a consequence, rising spreads can result in a well-run Italian firm paying higher interest rates than a poorly managed German competitor, calling into question the very idea of the single market as a mechanism to increase efficiency and productivity (Dullien, 2012; Dullien et al. 2020). This problem will be exacerbated to the extent to which some countries' ability to support potentially viable companies is constrained by fiscal limits whereas other countries are free to do so.

Furthermore, as many euro area banks still have a strong bias towards domestic sovereign debt, diverging sovereign yields pose a threat to financial stability. Especially in Italy's case, not only do higher financing costs weaken the scope for public spending, but also the lower price of government bonds stresses bank balance sheets. Increasing uncertainty and higher private-sector financing costs may further weaken the economy, leading to a surge in non-performing loans in the banking system.

## A common debt instrument

These are the reasons why a growing number of economists have called for the introduction of common instruments that provide fiscal support to member states, enabling them to weather the crisis without dramatic increases in public debt. While the proposals – such as coronabonds (Bofinger et al., 2020) or the Pandemic Solidarity Instrument (Grund et al., 2020) – differ in the amount of detail they provide and in their specific features, they all share the idea of common debt issued to finance crisis-related expenditure. For example, Bofinger et al. (2020) propose that the euro countries issue joint bonds with a volume of €1,000 billion to provide support to countries in the coronavirus crisis. The borrowed funds are distributed to them by a formula reflecting the severity of the health and economic crisis caused by COVID-19. Servicing and repayment could be organised by contributions to a repayment fund based on GDP or the ECB capital key. Liability for these bonds would be joint and several.

Proponents of such proposals argue that, as the new debt is supranational, not national in nature, financial markets would not see this increased debt as a direct increase in the liabilities of already fiscally weak member states. As the newly issued debt would cover a significant share of the additional borrowing needed by member states over the acute crisis period, they would not have to tap capital markets, insulating them from an increase in their spreads.

Finally, as coronabonds would be at least as safe as German sovereign bonds and have a significant volume, they would provide a European safe asset to the financial system at a time of increased uncertainty and could be used by the ECB for liquidity operations. Such a safe asset would act as a stability anchor for the financial system. Once market participants have confidence in its fundamental safety, its counter-cyclical price movements in crisis situations will have a stabilising effect on the economy throughout the euro area. Investors' flight to safety lowers financing costs, increasing the fiscal space, while higher prices improve banks' balance sheets (Tober, 2013). A large and liquid market of safe sovereign bonds in the euro area would furthermore bolster the international role of the euro, thereby benefiting the economies of all euro countries and increasing the international political sway of the EU (Theobald and Tober, 2018).

### Coronabonds vs. eurobonds

Even in Germany, the current proposals have been promoted by economists who in the past have not been vocal supporters of joint debt at the European level such as the eurobonds[1] discussed already during the euro crisis.[2] Even traditionally sceptical economists have underlined that the case for coronabonds is very different than that for eurobonds. In their eyes, coronabonds do not carry the risk of

1 Here, eurobonds refer to debt elements with joint and several liability to finance normal expenditure of member states' governments irrespective of the proposed constructions' issuance limits or rules.

2 Examples include Michael Hüther from the German Economic Institute (IW) or Gabriel Felbermayr from the Kiel Institute for the World Economy, both co-authors of Bofinger et al. (2020).

moral hazard, which has often been spotlighted in the debate on eurobonds, especially in Germany. After all, coronabonds are proposed as a one-off joint issuance for costs already incurred by an external event (the spread of the pandemic).

Despite the growing support for coronabonds by economists across the political spectrum, the idea has yet to win sufficient political support. While the Netherlands has been the most vocal opponent of the idea, supported by Austria and Finland, it is safe to say that, ultimately, the opposition of the German government has been decisive in blocking the introduction of coronabonds.

### Inadequacy of emergency support measures

The German finance ministry has argued that the support measures already decided – at least in principle – at the EU level could support the euro area economies sufficiently well. In this respect, three measures have been highlighted by the German government:

- On top of a €40 billion package announced in March, the European Investment Bank (EIB) has proposed offering additional guarantees to companies. In the recent statement from the Eurogroup Finance Ministers, a pan-European guarantee fund of €25 billion is mentioned that could support an additional €200 billion of EIB lending with a focus on SMEs.
- The European Commission has proposed a scheme to provide financial support for national short-time working (or similar) schemes, called SURE (temporary support to mitigate unemployment risks in an emergency). The programme is supposed to have a maximum volume of €100 billion. It will require member states to provide guarantees rather than capital upfront. Funds would be disbursed as loans.
- Lending by the European Stability Mechanism (ESM) will be available to all euro area member states up to 2% of GDP. This lending will not be subject to detailed conditionalities. However, the new credit line (Pandemic Crisis Support) is limited in time – up to when the COVID-19 crisis is over, with no clear indication of how that will be determined – and to "direct and indirect healthcare, cure and prevention related costs" (Eurogroup, 2020).

Moreover, there has been an agreement in principle on a EU recovery fund (ERF), but it has not been spelled out in detail and little is known about its likely volume, the allocation of funds between loans and grants, the refinancing procedure or its legal form. All that seems certain is that the recovery fund will be accompanied by a temporary increase in the EU budget.

Initially leaving the ERF aside for that reason, it is easy to see why the emergency measures cannot have a supportive effect equivalent to the discussed coronabonds. EIB loans, loans from the SURE fund and ESM loans are all credit-based and increase national debt by the amount of funds disbursed. Public finances will only be relieved to the extent that these loans are cheaper and/or of longer duration than those available on the market. Furthermore, in a situation in which local lenders and national promotional institutions have to be involved for the full lending capacity of the EIB programme, to achieve a leverage ratio of 8% seems at least optimistic. At the end of the day, the programme might largely merely reclassify existing national funds. The ESM credit line, which can provide significant interest savings, threatens to stigmatise those member states that use the funds, despite all political assurances, worsening their capital market conditions. While details of loan conditions for SURE are still sketchy, for as long as the ECB stands ready to limit yield spreads within narrow margins via PEPP and other measures, it is hard to see how the fiscal support can be macroeconomically significant, even for the hardest-hit countries.

With increased national debt levels and a de facto seniority of the credit lines from the EU schemes, there is no reason why investors should be less fickle than without these measures. The risk of a self-fulfilling fiscal crisis caused by increasing spreads of highly indebted euro countries thus remains basically unchanged and is limited only by the willingness and the ability of the ECB to act.

Returning to the ERF, it is easy to conceive how it could be set up in a way that it serves as an equivalent of the above-discussed coronabonds. In such a scenario, the EU would borrow at least €1,000 billion to fill the fund based on national guarantees, support national budgets from this fund, and service the debt from higher contributions to the EU budget or by new EU own resources. As at the moment, the Multiannual Financial Framework of the EU for the years 2021-2027 is being renegotiated, in principle, the EU budget's size could be raised accordingly.

The joint French-German initiative for a recovery fund of €500 billion would be a step in the right direction. According to the plan, the EU would tap capital markets and fund spending in the most affected sectors and regions in line with European priorities (Macron and Merkel, 2020). Although the actual volume of transfers between countries is likely to be relatively small, it is the joint borrowing and the frontloading that are important as they signal European unity and determination to financial markets and to citizens. Ideally, the design of the Fund could serve as a blueprint for more active fiscal cooperation in the future, which is essential for the longer-run stability of the monetary union.

It remains be seen whether the proposal will become reality. In some European countries, there is strong resistance to increasing the transfer element in the EU budget, even to the limited extent proposed by the French-German initiative. In particular, it is as yet unclear whether the so-called 'frugal' countries will agree to the coronabonds-like construction of the ERF, although the increased stability of the EU would also be in their longer-term interest.

## Endowing the EU with stabilising tools

In conclusion, the coronavirus crisis has made the euro area vulnerable to a replay of the sovereign debt crisis of the beginning of the past decade. The European Council has so far failed to come up with a decisive solution that breathes European solidarity and presents a common front to its financial markets as well as unity to its citizens. The ECB is being relied upon to do the heavy lifting. This is a hugely risky strategy. The European Parliament has called for the setting up of a sizable recovery fund. The European Commission and the heads of state and government need to seize the opportunity to tackle the serious threat to the integration project and endow the EU with tools to stabilise the economy in the current and future crises and to address longer-term challenges.
